# The association of prior paracetamol intake with outcome of very old intensive care patients with COVID-19: results from an international prospective multicentre trial

**DOI:** 10.1186/s12877-022-03709-w

**Published:** 2022-12-27

**Authors:** Philipp Heinrich Baldia, Bernhard Wernly, Hans Flaatten, Jesper Fjølner, Antonio Artigas, Bernardo Bollen Pinto, Joerg C. Schefold, Malte Kelm, Michael Beil, Raphael Romano Bruno, Stephan Binnebößel, Georg Wolff, Ralf Erkens, Sviri Sigal, Peter Vernon van Heerden, Wojciech Szczeklik, Muhammed Elhadi, Michael Joannidis, Sandra Oeyen, Brian Marsh, Finn H. Andersen, Rui Moreno, Susannah Leaver, Dylan W. De Lange, Bertrand Guidet, Christian Jung, COVIP study group, Philipp Eller, Michael Joannidis, Dieter Mesotten, Pascal Reper, Walter Swinnen, Nicolas Serck, ELISABETH DEWAELE, Helene Brix, Jens Brushoej, Pritpal Kumar, Helene Korvenius Nedergaard, Ida Riise Balleby, Camilla Bundesen, Maria Aagaard Hansen, Stine Uhrenholt, Helle Bundgaard, Richard Innes, James Gooch, Lenka Cagova, Elizabeth Potter, Michael Reay, Miriam Davey, Mohammed Abdelshafy Abusayed, Sally Humphreys, Arnaud Galbois, Cyril Charron, Caroline Hauw Berlemont, Guillaume Besch, Jean-Philippe Rigaud, Julien Maizel, Michel Djibré, Philippe Burtin, Pierre Garcon, Saad Nseir, Xavier Valette, Nica Alexandru, Nathalie Marin, Marie Vaissiere, Gaëtan PLANTEFEVE, Thierry Vanderlinden, Igor Jurcisin, Buno Megarbane, Benjamin Glenn Chousterman, François Dépret, Marc Garnier, Sebastien Besset, Johanna Oziel, Alexis Ferre, Stéphane Dauger, Guillaume Dumas, Bruno Goncalves, Lucie Vettoretti, Didier Thevenin, Stefan Schaller, Stefan Schaller, Muhammed Kurt, Andreas Faltlhauser, Stefan Schaller, Milena Milovanovic, Matthias Lutz, Gonxhe Shala, Hendrik Haake, Winfried Randerath, Anselm Kunstein, Patrick Meybohm, Stefan Schaller, Stephan Steiner, Eberhard Barth, Tudor Poerner, Philipp Simon, Marco Lorenz, Zouhir Dindane, Karl Friedrich Kuhn, Martin Welte, Ingo Voigt, Hans-Joachim Kabitz, Jakob Wollborn, Ulrich Goebel, Sandra Emily Stoll, Detlef Kindgen-Milles, Simon Dubler, Christian Jung, Kristina Fuest, Michael Schuster, Antonios Papadogoulas, Francesk Mulita, Nikoletta Rovina, Zoi Aidoni, EVANGELIA CHRISANTHOPOULOU, EUMORFIA KONDILI, Ioannis Andrianopoulos, Martijn Groenendijk, Mirjam Evers, Mirjam Evers, Lenneke van Lelyveld-Haas, Iwan Meynaar, Alexander Daniel Cornet, Marieke Zegers, Willem Dieperink, Dylan de Lange, Tom Dormans, Michael Hahn, Britt Sjøbøe, Hans Frank Strietzel, Theresa Olasveengen, Luis Romundstad, Anna Kluzik, Paweł Zatorski, Tomasz Drygalski, Jakub Klimkiewicz, Joanna Solek-pastuszka, Dariusz Onichimowski, Miroslaw Czuczwar, Ryszard Gawda, Jan Stefaniak, Karina Stefanska-Wronka, Ewa Zabul, Ana Isabel Pinho Oliveira, Rui Assis, Maria de Lurdes Campos Santos, Henrique Santos, Filipe Sousa Cardoso, André Gordinho, MJosé Arche Banzo, Begoña Zalba-Etayo, PATRICIA JIMENO CUBERO, Jesús Priego, Gemma Gomà, Teresa Maria Tomasa-Irriguible, Susana Sancho, Aida Fernández Ferreira, Eric Mayor Vázquez, Ángela Prado Mira, Mercedes Ibarz, David Iglesias, Susana Arias-Rivera, Fernando Frutos-Vivar, Sonia Lopez-Cuenca, Cesar Aldecoa, David Perez-Torres, Isabel Canas-Perez, Luis Tamayo-Lomas, Cristina Diaz-Rodriguez, Pablo Ruiz de Gopegui, Nawfel Ben-Hamouda, Andrea Roberti, Yvan Fleury, Nour Abidi, Alexander Dullenkopf, Richard Pugh, Sara Smuts

**Affiliations:** 1 Department of Cardiology, Pulmonology and Vascular Medicine, Medical Faculty, Heinrich-Heine-University Duesseldorf, Duesseldorf, Germany; 2 Department of Anaesthesiology, Perioperative Medicine and Intensive Care Medicine, Paracelsus Medical University, Salzburg, Austria; 3 Department of Clinical Medicine, Department of Anaestesia and Intensive Care, University of Bergen, Haukeland University Hospital, Bergen, Norway; 4 Department of Intensive Care, Aarhus University Hospital, Aarhus, Denmark; 5 Critical Care Centre, Sabadell Hospital University Institute Parc Tauli, Sabadell Barcelona, Spain; 6 Department of Acute Medicine, Geneva University Hospitals, Geneva, Switzerland; 7 Department of Intensive Care Medicine, Inselspital, Universitätsspital, University of Bern, Bern, Switzerland; 8 General & Medical Intensive Care Units, Hadassah Medical Center and Faculty of Medicine, Hebrew University of Jerusalem, Jerusalem, Israel; 9 Department of Intensive Care and Perioperative Medicine, Jagiellonian University Medical College, Krakow, Poland; 10 Faculty of Medicine, University of Tripoli, Tripoli, Libya; 11 Division of Intensive Care and Emergency Medicine, Department of Internal Medicine, Medical University Innsbruck, Innsbruck, Austria; 12 Department of Intensive Care 1K12IC, Ghent University Hospital, Ghent, Belgium; 13 Mater Misericordiae University Hospital, Dublin, Ireland; 14 Department Of Anaesthesia and Intensive Care, Ålesund Hospital, Ålesund, Norway. Dep. of Circulation and Medical Imaging, Norwegian University of Science and Technology, Trondheim, Norway; 15 Multipurpose and Neurocritical Intensive Care Unit, Hospital of São José, Central Lisbon University Hospital Centre, Lisbon, Portugal; 16 General Intensive Care, St George´S University Hospitals NHS Foundation Trust, London, UK; 17 Department of Intensive Care Medicine, University Medical Center, University Utrecht, Utrecht, Netherlands; 18 Institute Pierre Louis Epidemiology and Public Health, Medical Intensive Care Unit, Sorbonne University, UPMC, INSERM, Hôpital Saint-Antoine, Paris, France

**Keywords:** COVID-19, Frailty, ICU, Paracetamol, Analgesics

## Abstract

**Background:**

In the early COVID-19 pandemic concerns about the correct choice of analgesics in patients with COVID-19 were raised. Little data was available on potential usefulness or harmfulness of prescription free analgesics, such as paracetamol. This international multicentre study addresses that lack of evidence regarding the usefulness or potential harm of paracetamol intake prior to ICU admission in a setting of COVID-19 disease within a large, prospectively enrolled cohort of critically ill and frail intensive care unit (ICU) patients.

**Methods:**

This prospective international observation study (The COVIP study) recruited ICU patients ≥ 70 years admitted with COVID-19. Data on Sequential Organ Failure Assessment (SOFA) score, prior paracetamol intake within 10 days before admission, ICU therapy, limitations of care and survival during the ICU stay, at 30 days, and 3 months. Paracetamol intake was analysed for associations with ICU-, 30-day- and 3-month-mortality using Kaplan Meier analysis. Furthermore, sensitivity analyses were used to stratify 30-day-mortality in subgroups for patient-specific characteristics using logistic regression.

**Results:**

44% of the 2,646 patients with data recorded regarding paracetamol intake within 10 days prior to ICU admission took paracetamol. There was no difference in age between patients with and without paracetamol intake. Patients taking paracetamol suffered from more co-morbidities, namely diabetes mellitus (43% versus 34%, *p* < 0.001), arterial hypertension (70% versus 65%, *p* = 0.006) and had a higher score on Clinical Frailty Scale (CFS; IQR 2–5 versus IQR 2–4, *p* < 0.001). Patients under prior paracetamol treatment were less often subjected to intubation and vasopressor use, compared to patients without paracetamol intake (65 versus 71%, *p* < 0.001; 63 versus 69%, *p* = 0.007). Paracetamol intake was not associated with ICU-, 30-day- and 3-month-mortality, remaining true after multivariate adjusted analysis.

**Conclusion:**

Paracetamol intake prior to ICU admission was not associated with short-term and 3-month mortality in old, critically ill intensive care patients suffering from COVID-19.

Trial registration.

This prospective international multicentre study was registered on ClinicalTrials.gov with the identifier “NCT04321265” on March 25, 2020.

## Background

Older patients are more likely to die from COVID-19, the disease caused by Severe acute respiratory syndrome coronavirus type 2 (SARS-CoV-2) [1]. Early studies of COVID-19 have shown that especially old and frail patients are at particular risk for a worse outcome compared to younger people, with case fatality rates increasing up to 14.8% in patients aged 80 years and more. The majority of COVID-19 related deaths are in patients aged 80 years or greater [1–5]. These observations are in line with past studies of outcome of frail intensive care unit (ICU) patients showing that frailty – and not old age alone—is an important predictor of outcome in critically ill patients [6, 7]. In response to the eminently high vulnerability of old and frail patients, many countries chose to prioritize this vulnerable group in their vaccination programs to protect them from a likely fatal outcome [8]. This patient cohort is also of particular interest since they frequently take inter-current medications and have comorbid conditions.

In the early phase of the COVID-19 pandemic there were warnings not to use prescription-free analgesics such as ibuprofen and other non-steroidal anti-inflammatory drugs (NSAIDs), since they were suspected to cause higher morbidity and mortality. Those statements were taken up by the media and led to widespread confusion and fear of – up to then – commonly used drugs for everyday ailments, like headaches and fever [9, 10].

Several studies suggested a potential influence of NSAIDs on the clinical course of respiratory viral diseases, namely worsening the overall outcome due to a suppression of the initial inflammatory cascade [11, 12]. Despite an early focus on a potential detrimental effect of NSAIDs on clinical outcomes, studies have shown no association of NSAID use with mortality [12, 13]. However, the role and influence of paracetamol, often used as an alternative to NSAIDs, remains unclear. To date, no data are available on the effects of paracetamol intake on COVID-19 disease in the vulnerable cohort of very old and critically ill patients admitted to ICUs.

This international multicentre study addresses this lack of evidence regarding the usefulness or potential harm of paracetamol intake prior to ICU admission in a setting of COVID-19 disease within a large, prospectively enrolled cohort of critically ill and frail ICU patients.

## Methods

### Design and settings

This international multicentre study is part of the Very old Intensive care Patients (VIP) project and has been endorsed by the European Society of Intensive Care Medicine (ESICM, http://www.vipstudy.org). Furthermore, it was registered on ClinicalTrials.gov (ID: NCT04321265) and planned in adherence to the European Union General Data Privacy Regulation (GDPR) directive, which is implemented in most participating countries. The COVIP (COVID-19 in very old intensive care patients) investigation aims to improve and enhance the knowledge about relevant factors for predicting mortality in older COVID-19 patients to help detect these patients early on and prevent a worse outcome. National coordinators were responsible for ICU recruitment, coordination of national and local ethical permissions and supervision of patient recruitment, as in the previous VIP studies [14, 15]. Ethical approval was mandatory for study participation. In most of the countries informed consent was obligatory for inclusion. However, due to a waiver of informed consent by some local Ethics committees, in a few countries, recruitment was possible without informed consent as previously described [16]. Overall, 138 intensive care units from 28 countries participated in the COVIP study [5, 17]. A list of all collaborating centers is given in the acknowledgement section.

### Study population

COVIP recruited patients with proven COVID-19, defined as a positive polymerase chain reaction (PCR) test result, aged 70 years or older who were admitted to an ICU. Data collection started at ICU admission. Thus, data about pre-ICU triage were not available. The admission day was defined as day one, and all consecutive days were numbered sequentially from the admission date. The dataset contained patients enlisted to the COVIP study from 19^th^ March 2020 until the 4^th^ of February 2021.

### Data collection

All study sites used a uniform online electronic case report form (eCRF). For this subgroup analysis, only patients with documented information regarding intake of paracetamol up to ten days prior to ICU admission were included.

### Paracetamol intake

Paracetamol intake was defined as any oral or intravenous intake regardless of dosage and duration within the ten days prior to ICU admission, including during prior hospitalization, as reported by patient interviews; in case the patient was not able to respond, the relatives were asked to provide information. No differentiation was made between regular or irregular, i.e., single use, in reporting paracetamol intake.

The Sequential Organ Failure Score (SOFA) on admission was recorded [14, 15]. For calculation of the Horowitz Index (p_a_O_2_/FiO_2_-ratio), the first arterial blood gas analysis after ICU admission was used ideally within one hour of ICU admission. Additionally, the need for non-invasive (NIV) or invasive ventilation, prone positioning, tracheostomy, vasopressor use, renal replacement therapy (RRT) and limitation or withdrawal of life-sustaining therapy during the ICU stay were recorded.

### Data storage

The eCRF was constructed with the REDCap software [18]. Data storage and hosting of the eCRF took place on a secure server of Aarhus University in Denmark. The servers were operated in collaboration between the Information Technology Department and the Department of Clinical Medicine of the Aarhus University.

### Frailty assessment

The frailty level prior to the acute illness and hospital admission was assessed using the Clinical Frailty Scale (CFS) as described previously [14, 15]. Patients were grouped into three categories: fit (CFS 0–3) vulnerable (CFS 4) and frail (CFS ≥ 5).

### Statistical analysis

Continuous data were described as median ± interquartile range (IQR). Differences between independent groups were calculated using the Mann Whitney U-test. Categorical data were expressed as percentages. Kaplan–Meier analysis was used for assessment of mortality. For calculating differences between groups, the chi-square test was applied. Univariate and multivariate logistic regression analyses were performed to assess associations of paracetamol use with ICU-, 30-days and 3-months-mortality. We report (adjusted) odds ratios (OR) with respective 95% confidence intervals (CI). We performed sensitivity analyses plotting univariate OR and 95%CI. All tests were two-sided. A p-value of < 0.05 was considered statistically significant. Stata 16 was used for all statistical analyses (StataCorp LLC, 4905 Lakeway Drive, College Station, Brownsville, Texas, USA).

## Results

### *Demographic data* (age, sex, frailty, co-morbidities)

The study included 2,646 patients in total, of which 1,480 patients (56%) did not take paracetamol up to ten days prior to admission to an intensive care unit, whereas 1,166 patients (44%) confirmed paracetamol intake prior to ICU admission. The median age was 75 years in both groups (IQR 72–79 years, p > 0.98); significantly more women reported an intake of paracetamol 10 days prior to ICU admission. Patients on paracetamol intake were slightly more frail in comparison to patients without paracetamol intake (IQR 2–5 versus 2–4, *p* < 0.001). In addition, patients using paracetamol had more co-morbidities, such as arterial hypertension and diabetes mellitus in comparison to non-users (43% versus 34%, *p* < 0.001 for diabetes mellitus and 70% vs 65%, *p* = 0.006 for arterial hypertension). Concerning the occurrence of COVID-19 symptoms, patients under treatment with paracetamol had a shorter duration from symptom onset until ICU admission in comparison with patients without paracetamol medication (6 versus 7 days, *p* = 0.01). Additional data regarding patient demographics and co-morbidities are displayed in Table 1.

**Table 1:** Patient characteristics

	No paracetamol intake *(n* = *1480)*	paracetamol intake *(n* = *1166)*	*p*-value
Male sex (n)	73% (1076)	65% (753)	** < 0.001**
Age in years	75 (72–79)	75 (72–79)	0.98
70–79 years (n)	80% (1178)	78% (908)	
80–89 years (n)	19% (286)	21% (248)	
> 90 years (n)	1% (16)	1% (10)	
BMI	27 (25–31)	28 (25–31)	0.93
SOFA Score	5 (3–8)	5 (3–8)	**0.004**
CFS	3 (2–4)	3 (2–5)	** < 0.001**
Comorbidities			
Diabetes mellitus	34% (495)	43% (495)	** < 0.001**
Arterial hypertension	65% (957)	70% (814)	**0.006**
CAD	24% (350)	25% (286)	0.61
Chronic heart failure	14% (210)	15% (177)	0.47
Pulmonary disease	23% (333)	22% (258)	0.91
CKD	16% (235)	18% (213)	0.097

*Abbreviations: BMI* Body mass index (kg/m^2^), *CAD* Coronary artery disease, *CFS* Clinical Frailty Scale, *CKD* Chronic kidney disease, *IQR* Interquartile range, *SD* Standard deviation, *SOFA* Sequential Organ Failure Assessment

Categorical variables displayed as % (n), continuous variables as median (IQR). Patients with reported paracetamol intake had a higher SOFA Score on ICU admission than those without paracetamol intake

### Treatment in intensive care units

We observed a significant difference in SOFA scores on ICU admission between the two groups: those who received paracetamol treatment were admitted with a slightly higher SOFA score compared to those without paracetamol intake (5 versus 5, IQR 3–8 versus 3–8; cf. Table 1; *p* = 0.004). Furthermore, we observed several differences in intensive care treatment: Patients on paracetamol treatment prior to ICU admission were significantly less often subjected to endotracheal intubation and vasopressor treatment (65% versus 71%, *p* < 0.001 and 63% versus 68%, *p* = 0.007, respectively). No difference in tracheostomy rates were observed (16% versus 19%, *p* = 0.073). Additionally, the need for renal replacement therapy and NIV did not differ between both groups (14% versus 15%, *p* = 0.3; 26% vs 27%, *p* = 0.58, respectively). Concerning treatment withholding and withdrawal, patients with a reported paracetamol intake were less likely to be subjected to either treatment withholding or withdrawal, such as discontinuing respiratory or circulatory support, when compared to those without paracetamol intake (25% versus 29%, *p* = 0.021; 16% versus 19%, *p* = 0.011, respectively).

### Mortality

**Fig. 1:**
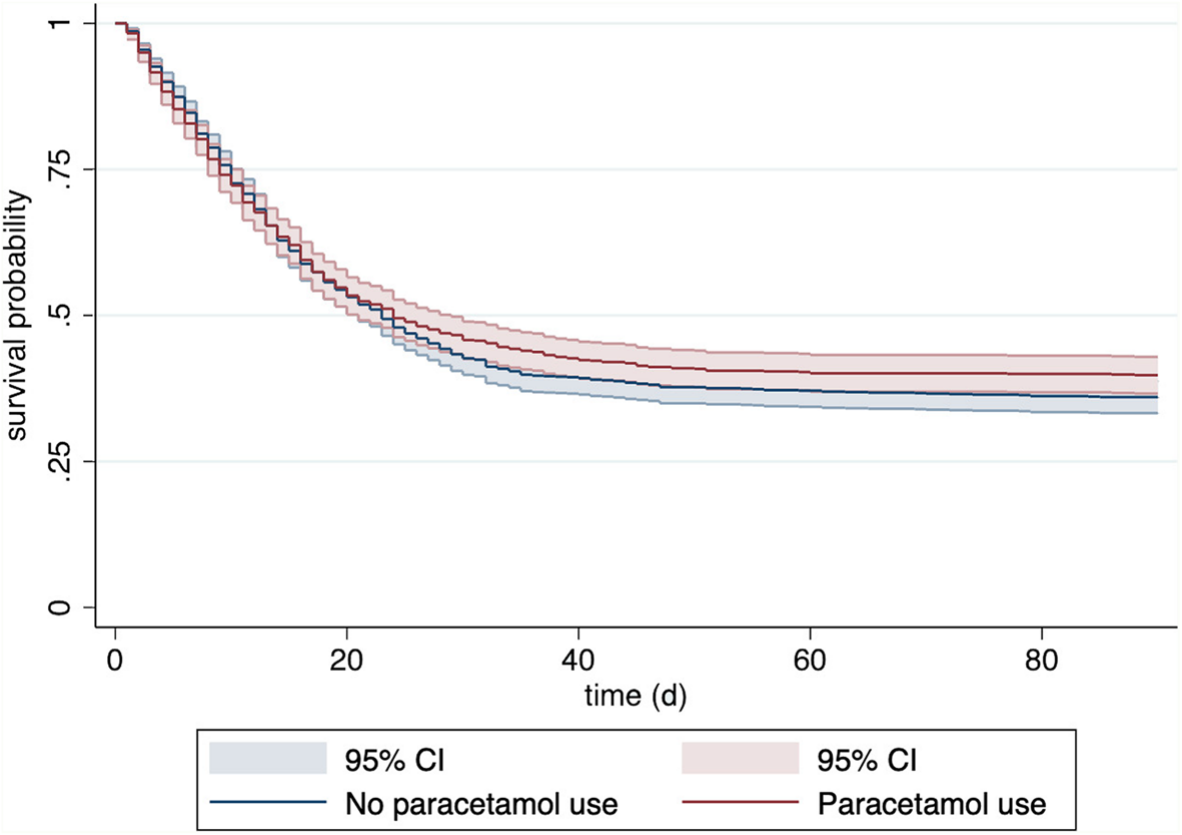
Kaplan–Meier curve for patients with (red) and without (blue) paracetamol intake prior to up to ten days before admission to intensive care units for COVID-19 (with 95% confidence interval)

No difference in mortality was observed between patients with and without paracetamol intake up to ten days prior to ICU admission (Fig. 1): ICU mortality was 46% vs 48% (*p* = 0.3) in patients with and without paracetamol intake respectively; 30-day mortality was 48% versus 51% (*p* = 0.12) in patients with and without paracetamol intake; 3-month mortality rates were 60% versus 64% (*p* = 0.059), respectively.

**Fig. 2:**
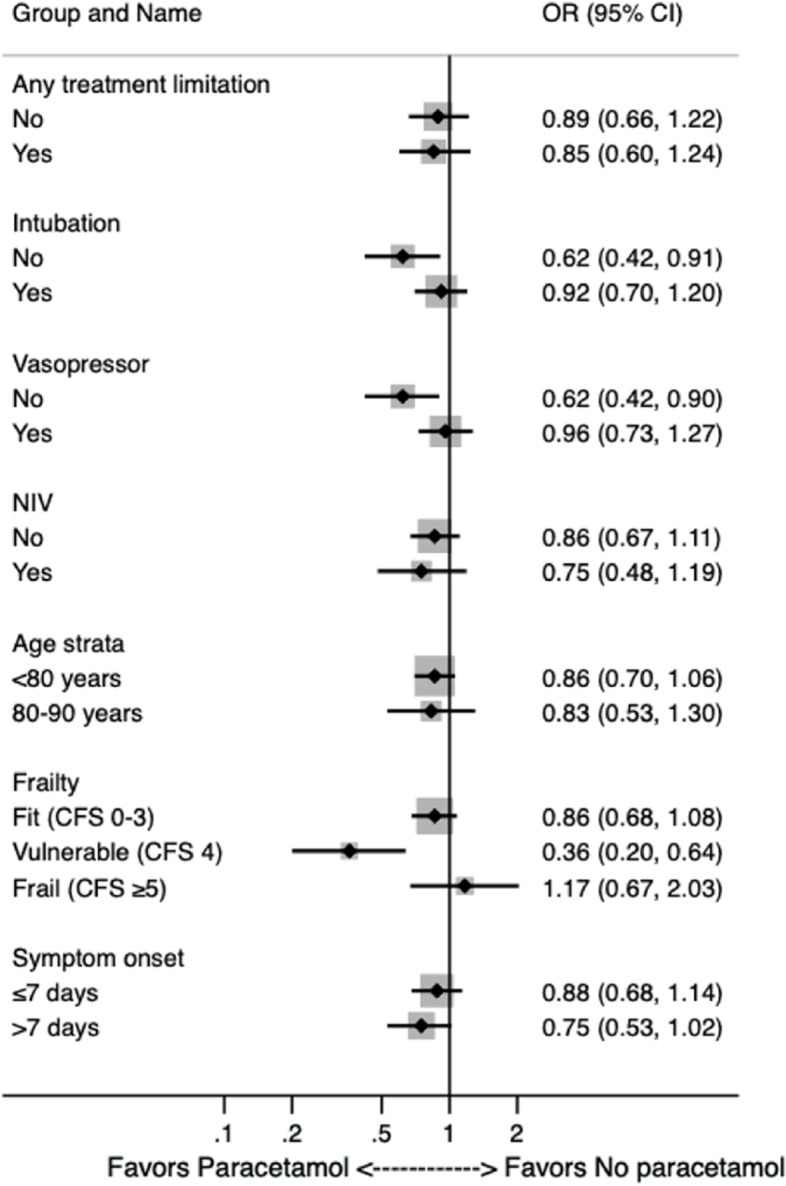
Sensitivity analyses stratifying 30-day-mortality in subgroups for patient-specific characteristics using logistic regression producing univariate odds ratios. The 30-day-mortality was similar between patients with and without paracetamol intake regardless of treatment limitations, the use of NIV, age strata and the time from symptom onset until admission. Patients categorized as vulnerable according to CFS (OR 0.36) and without endotracheal intubation and vasopressor use (OR 0.62, respectively) were more likely to take paracetamol

Sensitivity analyses stratifying 30-day-mortality into subgroups for patient-specific characteristics using logistic regression producing univariate odds ratios are shown in Fig. 2. The 30-day-mortality was similar between patients with and without paracetamol intake regardless of treatment limitations, the use of NIV, age strata and the time from symptom onset until admission. There was a trend towards higher mortality in patients with paracetamol intake without intubation and in vulnerable patients as assessed by CFS.

After adjustment for age, sex, SOFA score and CFS at admission, paracetamol use was not associated with ICU (aOR 0.93 95%CI 0.78–1.11; *p* = 0.42), 30-day (aOR 0.86 95%CI 0.72–1.03; *p* = 0.10) and 3-month (aOR 0.88 95%CI 0.72–1.07; *p* = 0.20) mortality.

## Discussion

In this subgroup analysis of critically ill patients ≥ 70 years of age, we investigated the influence of prior paracetamol intake on short-term and long-term mortality in patients with COVID-19. At the beginning of the pandemic, on the 14th of March 2020, the French Ministry of Health published data on 400 patients suffering from a severe clinical course of COVID-19 after taking ibuprofen; it therefore advised against the use of ibuprofen and other NSAIDs as the analgesics and antipyretics of choice. Instead, several health experts advised the use of paracetamol in case of fever or pain [9, 19, 20]. Previous studies investigated possible pathophysiological mechanisms by which NSAIDs and paracetamol could influence the clinical course of an infection with SARS-CoV-2 and other respiratory viruses [12, 21–23].

Paracetamol on the other hand is not included in the group of NSAIDs and has a more pronounced effect on cyclooxygenase (COX) 3 iso-enzyme, which is located in the central nervous system in contrast to the COX variants 1 and 2; thus, paracetamol has more central antipyretic and analgesic effects without compromising the systemic inflammatory cascade [24, 25]. Our findings are in line with current literature, confirming the safety of paracetamol as a potent analgesic and antipyretic drug in viral infections and especially in the case of COVID-19 disease [26, 27]. This is even true in a setting of critically ill and vulnerable, very old and frail people admitted to an ICU. We found no association of paracetamol intake prior to ICU admission with either ICU-, 30-day- and 3-months-mortality in patients with COVID-19 aged 70 years or more.

Since the study at hand did not report on analgesic use other than paracetamol, e.g., aspirin or NSAIDs, it remains unclear, whether paracetamol has any specific impact on the clinical course concerning the extent of intensive care treatment. Additionally, no differences in renal replacement therapy were observed. This may be explained by the mainly hepatic metabolism of paracetamol and a central activation of the COX 1 splice variant COX 3 as stated above [11, 24, 25, 28].

Patients on paracetamol treatment prior to hospitalization were also less prone to treatment withholding or withdrawal in comparison with those without paracetamol intake. In the light of the increased frailty of the paracetamol group, it remains unclear, why, despite this difference in co-morbidities, such as arterial hypertension and diabetes, they were less likely to have treatment withheld and withdrawn. This may be the result of the heterogeneity due to the international and multicentric setup of the COVIP study; hence individual differences, ethical, socio-medical patient backgrounds and the current epidemiological local burden of COVID-19 disease in the study sites must be considered, when discussing the observed differences in therapy and therapeutic limitations.

Our results are in line with findings by Park and colleagues, who showed safety of paracetamol in comparison to ibuprofen regarding the outcome of COVID-19 disease by analysing a propensity matched cohort of Korean patients in the first wave of the SARS-CoV-2 pandemic [29]. Similar results were published by Rinott et al. in a retrospective study on Israeli patients with a median age of 45 years, who reported ibuprofen or paracetamol intake up to 14 days prior to study inclusion; no difference in mortality or respiratory support rates were observed [30]. Further data on safety of paracetamol and NSAIDs were provided by Chandan and colleagues in a retrospective propensity matched study of 17,190 patients with osteoarthritis in the United Kingdom, who were prescribed either paracetamol and codeine, paracetamol and dihydrocodeine or NSAID: the study showed no increased risk of COVID-19 disease or mortality in both groups [31].

Importantly, we do neither observe paracetamols efficacy nor its safety in use. In summary, our multi-centre study suggests safety of paracetamol intake prior to hospitalization in intensive care units in a vulnerable and frail collective of more than 70 years old patients suffering from COVID-19 disease. This hypothesis is based on the evidence provided within this international, prospective multicentre study.

### Limitations

Our study has some methodological limitations. Firstly, the study lacks a control group of younger patients admitted to ICU wards for severe COVID-19. Secondly, no control group of non-ICU patients age ≥ 70 was analyzed. Furthermore, data recording was only limited to time after ICU admission, thus leaving out information on pre-ICU care and possible triage. In addition, no detailed information on ICU equipment, quality of care, nurse-patient ratios and measure of staff stress was obtained; neither was paracetamol use during ICU treatment addressed. These local circumstances may affect the care of older ICU patients [32]. Also, participating countries varied widely in their care structure, thus resulting in a large heterogeneity in the level of care and the regional burden of care regarding local COVID-19 cases. Regarding paracetamol intake, we did not assess and document the doses of paracetamol ingested. Limitations of care in the pandemic surges did not allow to measure plasma metabolites and estimate true drug exposure, thus limiting data reliability. Lastly, our study did not analyze the intake of non-steroidal anti-inflammatory drugs, such as ibuprofen or aspirin and their corresponding impact on mortality and clinical course. Therefore, no recommendation towards a specific subgroup of analgesics can be made.

## Conclusion

In the international multicentre COVIP study of old, critically ill patients with COVID-19, we found no association of paracetamol intake prior to ICU admission with short-term and 3-month mortality. Paracetamol is therefore likely safe for analgesic and antipyretic use in this group.

## Data Availability

Individual participant data that underlie the results reported in this article are available to investigators whose proposed use of the data has been approved by the COVIP steering committee. The anonymized data used and analyzed in this study can be requested from the corresponding author on reasonable request if required.

## Abbreviations

BMI: Body mass index (kg/m^2^)
CAD: Coronary artery disease
CFS: Clinical Frailty Scale
CI: Confidence interval
CKD: Chronic kidney disease
COVID-19: Coronavirus disease 2019
COX: Cyclooxygenase
eCRF: Electronic case report form
FiO_2_: Fraction of inspired oxygen
ICU: Intensive care unit
IQR: Interquartile range
NIV: Non-invasive ventilation
NSAID: Non-steroidal anti-inflammatory drug
(a)OR: (Adjusted) odds ratio
p_a_O_2_: Arterial partial oxygen pressure
RRT: Renal replacement therapy
SARS-CoV-2: Severe acute respiratory syndrome coronavirus type 2
SD: Standard deviation
SOFA: Sequential Organ Failure Score
